# Dance behaviour in cockatoos: Implications for cognitive processes and welfare

**DOI:** 10.1371/journal.pone.0328487

**Published:** 2025-08-06

**Authors:** Natasha Lubke, Suzanne D.E. Held, Melanie Massaro, Rafael Freire

**Affiliations:** 1 School of Agricultural, Environmental and Veterinary Sciences, Charles Sturt University, Elizabeth Mitchell Drive, Thurgoona, New South Wales, Australia; 2 School of Veterinary Sciences, Bristol University, Langford House, North Somerset, United Kingdom; University Centre Sparsholt, UNITED KINGDOM OF GREAT BRITAIN AND NORTHERN IRELAND

## Abstract

Parrots (Aves, *Psittaciformes*) in captivity have been reported to show dance behaviour in response to music, which may involve complex cognitive processes including imitation, vocal learning and entrainment. Dance behaviour in parrots may be indicative of a positive welfare state raising the possibility of using music as a form of environmental enrichment. In this study we studied dance behaviour in cockatoos (*Cacatuidae*) through an online video study and a playback experiment. First, we identified and defined cockatoo dance movements to music from videos posted on social media to reveal the extent of this behaviour in different species. Second, to test whether music elicited dance behaviour we conducted a preliminary playback experiment on captive cockatoos, whereby birds were presented with periods of music playback, no audio playback and an audio podcast. From 45 online videos representing five different cockatoo species we identified and described 17 new dance movements. We also found 17 rare movements observed in only one bird and not previously reported in the literature, which in many cases consisted of combinations of different movements. A cluster analysis indicated that inter-species similarities in dance movements were not related to phylogenetic relatedness. In the playback study, which involved zoo-housed male-female pairs of three species of cockatoos, all birds in all treatments showed dance behaviour but there was no significant effect of treatment on the probability of showing dancing behaviour. We conclude that dance behaviour in cockatoos is composed of a wide range of different movements and further research would be beneficial to determine if music can trigger dance in captive birds and serve as a form of environmental enrichment.

## Introduction

Anecdotally, parrots (*Psittaciformes*) have been reported to show “dancing” behaviour to music in captivity which has been supported by studies on a few individuals. Schachner et al. [1] and Patel et al. [2] exposed an African grey parrot (*Psittacus erithacus*) and a sulphur-crested cockatoo (*Cacatua galerita eleanora)*, respectively, to novel music stimuli and found that both birds displayed spontaneous periodic movement with head bobbing and one bird also displayed periodic foot tapping. In addition, Keehn et al. [3] studied the same captive sulphur crested cockatoo as Patel et al. [2], named Snowball, who danced to music spontaneously. Both Keehn et al. [3] and Patel et al. [2] also reported dance behaviour in other parrots, indicating that dance behaviour is not limited to just a few individuals though questions remain as to the source behaviour and function of dance behaviour in parrots. In addition, dance behaviour may be indicative of a positive welfare state and music may serve as a form of environmental enrichment.

In the wild, some birds appear to be able to synchronise motor output (e.g., tapping, bobbing and stepping) to their own, internally created beat, as part of complex courtship displays which have been described as dancing. For example, both sexes of the blue-capped cordon-bleu (*Uraeginthus cyanocephalus)* perform complex courtship displays that include a combination of motor behaviours coordinated with their songs [4]. During courtship, the male palm cockatoo (*Probosciger aterrimus*) uses a modified stick or seedpod to strike a hollow tree limb repeatedly and this drumming is accompanied by vocal and visual displays directed towards females [5]. The analysis of the beat sequences demonstrated that they were marked with a low variance in interbeat intervals and were highly regular and predictable, which is a key feature of human music [5]. Sexual selection has likely led to the evolution of rhythmic courtship displays in wild birds as these courtship displays clearly have the adaptive function to attract mates.

However, to date it remains unclear why parrots show dance behaviour in response to music in captivity when birds are not courting or in the absence of any potential sexual partner. The ability to dance in parrots may be related to their exceptional vocal learning [6] and vocal mimicry abilities [1,7]. Vocal learning is associated with evolutionary modifications to the forebrain, an area that also appears to play a key role in mediating a link between auditory input and motor output during learning [8]. In addition, the synchronization of body movement (e.g., foot tapping, head bobbing) to an externally perceived rhythm is referred to entrainment in biomusicology [1]. Entrainment involves the recognition of the regularity in auditory stimuli and the ability to adjust the motor output to the perceived rhythm [8]. Entrainment is the foundation of coordinated dance in humans [9] but is rarely seen in non-humans. Although it has been suggested that vocal mimicry is important for entrainment, not all vocal mimics have been observed to entrain to musical rhythm. Vocal mimics include humans, songbirds, parrots, hummingbirds, cetaceans, pinnipeds, elephants and some bats [1], yet of these only humans and parrots have so far shown evidence of spontaneous entrainment to musical rhythm.

Furthermore, parrots are long-lived and intelligent animals that form long-lasting relationships with conspecifics in the wild, and as a result, in captivity parrots often show poor welfare [10]. Hence, there is a need to better understand which housing and management interventions can improve their welfare in captivity [11]. Dancing to music can be performed repeatedly and appears self-rewarding and indicate of a positive welfare state, possibly indicating a form of locomotory play behaviour [12,13]. Animal welfare scientists are therefore interested in parrot dancing not only because it may indicate a positive welfare state, but also because music playback could be applied as a form of enrichment to enhance the welfare of captive birds [14]. For example, Robbins and Margulis [15] reported evidence of positive responses after three different African bird species were exposed to natural sounds, classical music and rock music. Studies on the effect of music on birds and parrots may therefore provide useful insights into indicators of positive welfare in birds, as well as practical environmental enrichment interventions to improve bird welfare.

Our research objective was to further understand dance behaviour in parrots and potentially provide a suitable paradigm to study bird cognition and positive welfare. Building on work by Keehn et al. [3] who characterised 14 dance movements, we used online videos to describe in detail dance movements to obtain a more complete picture of dance movements within and between cockatoo species. Our aim was to determine the ubiquitousness and forms of dance behaviour and examine inter-species similarities in dance behaviour. In addition, we conducted an experimental study on captive cockatoos in Wagga Wagga Zoo to test the hypothesis that music playback elicits dance behaviour in cockatoos.

## Materials and methods

### Part 1: Video study

#### Sample selection.

We searched YouTube (https://www.youtube.com/), Facebook (https://www.facebook.com/), Tik Tok (https://www.tiktok.com) and Instagram (https://www.instagram.com/) to find videos of parrots showing dance behaviour using the following search terms; “birds dancing”, “bird dance”, “birds dancing Elvis”, “parrots dancing”, “dancing Australian parrot”, “galah dance”, “bird dancing to rap music” and “bird dancing to rock music”.

These searches identified over a hundred videos which were subjected to an initial screening that involved watching the video for at least 10 seconds. The location, setting and visibility of the bird and variation in dancing was noted as well as checking that the music was being played to the bird at the time the video was made, and not overlaid on the video at a later stage. All videos in which the bird was not clearly visible (e.g., in a cage) or music was added to the video were excluded from further analysis.

After this initial screening, the remaining 83 videos were watched in full to determine their suitability for inclusion in further analysis. The species, duration, human engagement, music genre and preliminary dance movement observations were recorded. Videos had to meet the following criteria: 1) show a cockatoo (*Cacauidae*) in a domestic setting where music was played to the bird at the time the dance behaviour was observed, 2) the camera angle provided a good view of the parrot, and 3) the parrot performed at least two different dance movements. In addition, only one video from a particular channel or profile was selected to avoid obtaining multiple dance videos of the same individual parrot. To include a species in further data analyses, at least three suitable videos of a species had to be found. The 45 suitable videos of dance behaviour, showing an average dance duration of 70 ± 7.2s and represented 45 different parrots included in the data analysis are shown in the supplementary material (Supporting information, S1 Table).

#### Development of dancing ethogram and behavioural sampling.

We used the 16 dance movements described by Keehn et al. [3] as the basis for our ethogram and defined new movements. Behaviour was scored in two steps and involved multiple viewings at normal and slow-motion speeds. First, we scored the presence/absence of defined dance movements using one-zero sampling and used this part of the analysis to re-define and re-classify new dance movements. New dance movements were identified and included in a preliminary ethogram of parrot dance behaviour along with two examples of this movement. If a dance movement was displayed by only one parrot, it was recorded as a “rare” dance movement.

Second, we carried out another round of behavioural sampling at 0.75 playback speed when available to score the number of dance movements. The number of each movement were scored using the description and behavioural scoring protocol described in Supporting information, S2 Table. All videos were then watched a second time to ensure intra-observer reliability. During this round of scoring we identified 31 new variations of existing movements to create the final dance ethogram (S2 Table). Care was taken to use the language of Keehn et al. [3] to maintain consistency with emerging literature on this topic.

### Part 2: Preliminary experimental study

This research was approved by Charles Sturt University’s Animal Ethics Committee (approval number 2024EA-06). Two sulphur crested cockatoos (*Cacatua galerita*), two Major Mitchell cockatoos (*Lophochroa leadbeateri*) and two galahs (*Eolophus roseicapilla*) were studied at Wagga Wagga Zoo (Wagga Wagga, NSW) to test whether music elicits dance behaviour in cockatoos. The adult birds were of unknown age and origin and had been at this zoo for over two years and had never been exposed to music. The birds were paired within aviaries (one male and one female of the same species), and each of the three aviaries was approximately 8m x 4m, and 4m high and furnished with a range of branches, perches and foraging enrichments. They were fed a mixture of seeds and fresh fruit. Nest boxes were at the back of aviaries and the floor was covered with grass at the front and bark chips at the back, with logs and branches.

Three treatments were applied on nine days over a 6-week period on two days of the week when the zoo was closed to the public. Initial observations indicated that the birds were more active in the mornings, so the treatments were presented from 8:30am - 9:40am. The three treatments were: 1) audio playback of the song “The Nights” by Avicii (Music treatment), 2) no playback (Control treatment) and 3) audio playback of the podcast “She’s on The Money” (Podcast treatment). The music chosen, described as “progressive house”, has a beat of 126BPM and duration of 2.56 min and was played in a loop for 20 minutes. This song was chosen because it was similar to some of the music heard in videos in Part 1. It is unlikely that the birds had been exposed to this music before, though we are not certain, and it should be noted that the podcast treatment did not include any music. A Bluetooth speaker (JBL Flip 5, Harman International, Stamford, USA) was used for both audio playback treatments with the volume set to a human comfortable listening level in the cages. Each treatment was applied sequentially each day for 20 minutes with a 1-minute reset period between treatments to allow set up. Order of presentation of the treatments was determined by a Latin square design. Behavioural data was collected from the video recording using Sony camcorders (DCR SR45) on tripods 3m from each cage. The definitions of dance movements and scoring method was the same as for Part 1 (S2 Table).

### Statistical analysis

Data for Part 1 was obtained from publicly available videos, and raw data is available from https://figshare.com/account/items/28319255/edit. For Part 1, summary statistics was undertaken to provide the total of birds performing each dance movement, the total number of movements occurring, the average duration of each dance movement and the number of times an individual bird carried out the movement. To determine if there were species differences in dance behaviour, we first calculated the percentage of each species showing each dance movement. A hierarchical cluster analysis was then performed using SPSS (version 26.1, IBM statistics) to identify similarities and differences in dance movements between species.

For Part 2, the incidences of dance movements were rare with many zeros, which were not amenable to parametric analysis even with zero-inflated models. We therefore converted our variables of interest to two binomial variables; 1) whether dance movements were recorded or not during presentation of a treatment and 2) whether dance movements were seen or not in a sequence. Analyses of both variables was undertaken using a binary logistic regression with treatment and day as factors and bird identity as a random effect using R [16].

## Results

### Part 1: Video analysis

In total we found nine species of cockatoos dancing out of a total of 21 species occurring worldwide. We included 18 videos of dancing white cockatoos (*Cacatua alba),* 13 videos of Goffin’s cockatoos (*Cacatua goffiniana*), eight videos of sulphur crested cockatoo (*Cacatua galerita*) and three videos each of little corellas (*Cacatua sanguinea)* and Moluccan cockatoo *(Cacatua moluccensis)* (Table 1). We also found videos of dancing in palm cockatoo (*Probosciger aterrimus*, N = 1*),* long billed corella *(Cacatua tenuirostris*, N = 1*),* cockatiel *(Nymphicus hollandicus*, N = 1) and galah (*Eolophus roseicapilla*, N = 2), but due to the small sample size these videos were not included in our analyses. Various locations, music genres, number of birds, and presence of people was identified during video analysis. As seen in S1 Table, the most common setting in the videos was the birds on a chair (11), alone (32) and listening to pop music (14).

**Table 1:** Cockatoo species included in our analyses, number of videos/individuals and mean duration (with range) of dancing behaviour.

Species	Number of videos/individuals	Duration of dancing (seconds)
Goffins cockatoo (*Cacatua goffiniana)*	13	65 (11–149)
Little corella (*Cacatua sanguinea)*	3	156 (66–239)
Moluccan cockatoo (*Cacatua moluccensis*)	3	95 (52–137)
Sulphur crested cockatoo (*Cacatua galerita*)	8	61 (19–178)
White cockatoos (*Cacatua alba*)	18	53 (14–146)

**Fig 1:**
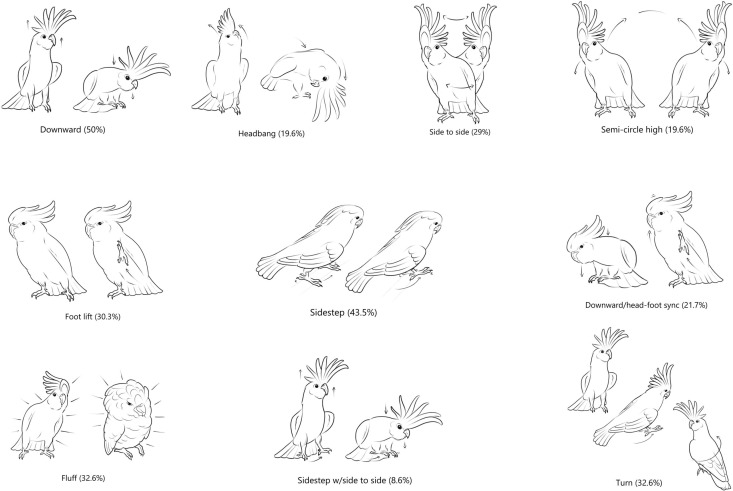
Illustration of the 10 most common recorded dance movements. Ethogram descriptors based on Keehn et al. [3] and illustrations by Zenna Lugosi.

In our sample of 45 videos/individuals we found 13 of the 16 dance movements described by Keehn et al. [3] We defined 17 new dance movements (S2 Table), for a total of 30 different dance movements reported in this study. It should be noted that we re-named the movement “counter-clockwise circle” described by Keehn et al. [3] as “Head Circle” since one bird rotated its head in a clockwise direction. We did not observe any instances of parrots doing the Vouge, Headbang with lifted foot or Headbang/Semi-Circle Low Interchanged dance movements identified by Keehn et al. [3]. A total of 17 rare movements displayed by only one parrot were recorded (S3 Table). The most common movement was “Downward” performed by 50% of the cockatoos, followed by Sidestep observed in 43% of videos. Illustrations of the 10 most common dance movements is provided in Fig 1.

The average duration of each movement was also calculated, and downward/head-foot sync with wings (30 s) and headbang with wings (113 s) took the most time (Table 2). The movement that was carried out for the shortest duration was headbang with stepping (Table 2). The least common movement combinations are headbang with stepping, flapping, and wing pump being performed by 4.3% of birds (Table 2). The most performed movements per individual bird was the mobile jump for 36.7 movements and downward with crest being performed 34.4 times per bird (Table 2).

**Table 2:** Dance movement analysis of the 17 new dance movements identified and number of parrots, percentage, total count and number of average movements per cockatoo.

Movement	Number of parrots (%)	Total count	Average movement per parrot
Headbang	9 (19.6)	137	17 (1–42)
Downward	23 (50)	534	23.9 (1–67)
Head Shake	6 (13)	10	1.7 (1–4)
Side to Side	18 (39)	233	12.9 (1–54)
Head circle	7 (15.5)	58	8.3 (1–38)
Head Downward and Shake	3 (6.5)	13	4.3 (2–7)
Semi-Circle Low	4 (8.7)	51	12.8 (3–28)
Semi-Circle High	9 (19.6)	53	5.9 (1–13)
Head Turn	4 (8.6)	72	18 (5–41)
Head Figure 8	5 (10.9)	74	14.8 (2–42)
Wings back	2 (4.3)	60	30 (13–47)
Flapping	2 (4.3)	14	7 (2–12)
Foot Lift	14 (30.4)	31	2.2 (1–5)
Sidestep	20 (43.5)	148	7.5 (1–30)
Downward/Head-Foot Sync	10 (21.7)	231	23.1 (2–60)
Downward Walk	10 (21.7)	78	7.4 (2–16)
Sidestep w/side to side	4 (8.6)	96	24 (8–62)
Body Roll	5 (10.9)	44	8.8 (2–18)
Pose	6 (13)	12	2 (1–3)
Fluff	15 (32.6)	26	1.7 (1–5)
Stationary Jump	4 (8.6)	42	10.5 (3–16)
Moving Jump	3 (6.5)	110	36.7 (12–85)
Turn	15 (32.6)	39	2.6 (1–7)
Jump Turn	2 (4.3)	8	4 (2–6)
Complex	5 (10.9)	6	1.5 (1–2)

**Fig 2:**
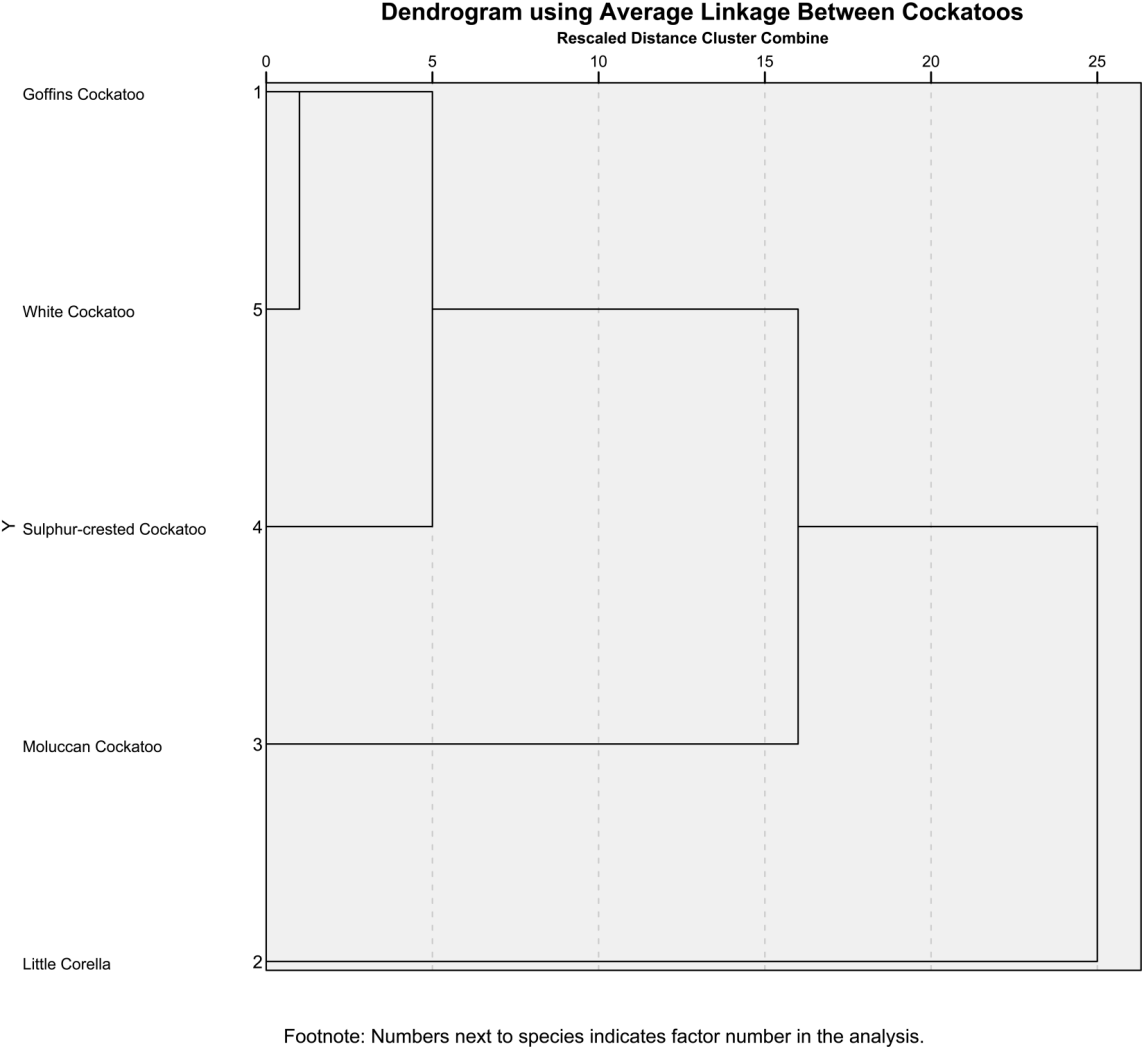
Cluster analysis of average presence and absence of dance movements for five cockatoo species.

Cluster analysis indicated that the dancing by Goffin’s cockatoo and White cockatoo was most similar, and little corellas differ the most to that of other cockatoos (Fig 2). The results show that each species has a unique top 10 common dance movements (S1 Fig). Among little corellas, Moluccan cockatoos, and Goffin’s cockatoos Side to Side, Downward, Foot Lift and Head Bang are all shared common movements. Typically, the common movements are the same between the species but are present at different frequencies. Sulphur crested cockatoos have three dance movements which are not shared with other species, these are Semi-Circle Low, Semi-Circle High with Crest and Head Foot sync (S1 Fig). The number of dance movements shown by each bird is shown in Supporting information (S2 Fig).

### Part 2: Preliminary Experimental Study

All birds showed dance movements on at least one day per treatment. One Galah (G2) was observed to dance the most in response to all treatments (Table 3). Dance movements in sequence, which are important to consider as repetition is an important feature of dancing, were seen in all birds in all treatments. Variation in dance movements is also important to consider and the six birds performed between 4 and 10 different movements in all treatments (Table 3).

**Table 3:** Summary of total single dance movements (Single), total movements in sequence (Sequence) and number of different movements (Different) recorded in the experimental study.

	Music treatment			Podcast treatment			Control treatment		
**Bird**	**Single**	**Sequence**	**Different**	**Single**	**Sequence**	**Different**	**Single**	**Sequence**	**Different**
SCC1 (M)	2	53	10	17	182	7	7	131	10
SCC2 (F)	20	33	5	21	76	10	18	7	5
MMC1 (M)	39	25	7	27	12	6	21	12	6
MMC2 (F)	52	22	6	29	18	4	85	7	4
G1 (M)	9	45	6	6	50	6	11	100	5
G2 (F)	30	257	9	12	187	7	18	187	8

SCC refers to Sulphur crested cockatoo, MMC to Major Mitchell cockatoo and G to Galah. M and F to sex of bird.

Binary logistic regression indicated no significant effect of treatment on the probability of showing at least one dance movement (χ^2^ = 1.73, df = 2, P = 0.42). Day (χ^2^ = 12.3, df = 8, P = 0.14) or the treatment/day interaction (χ^2^ = 11.92, df = 16, P = 0.75) did not significantly influence the probability of showing at least one dance movement (S3a Fig). Additionally, the probability of movements occurring in a sequence was not influenced by treatment (χ^2^ = 0.54, df = 2, P = 0.76), day (χ^2^ = 14.73, df = 8, P = 0.06) or treatment/day interaction (χ^2^ = 17.54, df = 16, P = 0.35; Supporting information, S3b Fig). Videos from Part 2 are available to other researchers upon request.

## Discussion

In summary, we found many examples of dance behaviour in a range of cockatoo species. From video analysis, we were able to construct an ethogram of 30 dance movements, and 17 rare movements performed by only one bird were also identified. Cluster analyses indicated that the Goffin’s cockatoo and white cockatoo appear to have the most similar movements and the Goffin’s cockatoo and little corella have the least similar movements. In a playback experiment (Part 2), all birds were found to show dance behaviour to all treatments, but there was no effect of music playback on the probability of showing dance movements either singly or in sequence.

The use of social media to access a large sample size has greatly built upon the findings of Keehn et al. [3] and increased our knowledge of the species that dance and the types of movements parrots utilise while dancing. However, it should be noted that although social media can provide some valuable insights into animal behaviour, it does have several limitations. First, although we excluded videos in which the music was clearly overdubbed, in some cases it was difficult to unequivocally determine if the music was being played to the birds. Second, online videos do not show or mention what was outside the video capture, so we cannot determine if events outside the video capture may have influenced the birds. Third, although research indicates that parrots can spontaneously develop dance behaviour [1], we are not able to determine if birds in the online videos had received any training. Lastly, it is important to note that to avoid bias in our sample we have, with a few disclosed exceptions, included all videos arising from our search. The result is that some videos may show parrots with sub-optimal welfare and the videos cited in this manuscript should not be considered as indicative of appropriate parrot care and management. Nonetheless, we found that dancing is present in nine species of cockatoos, and the dancing behaviour of 45 individual birds spanning five cockatoo species (Table 1) was further analysed. Out of 30 dance movements observed in multiple birds, and a further 17 rare movements observed in only one bird, Downward movement was the most common observed in 50% of birds. Overall, the movements involving just the head were more common and the least common movements were those involving just wings (i.e., Flapping and Wings Back). The new dance movements were identified and defined, and a scoring method was developed to support further research on this topic.

Dance behaviour is perhaps a more common behaviour in cockatoos than previously thought. We found through our video analysis dancing behaviour in nine species of cockatoos and in our playback experiment an additional species, the Major Mitchell cockatoo was also observed dancing, resulting in dancing behaviour being present in 10 out of 21 known species of cockatoos. The species similarities of dancing as revealed by a cluster analysis showed that the Goffin’s Cockatoo and white cockatoos are most similar, and Goffin’s cockatoo and little corella are the least similar when comparing dance movements. In contrast, a phylogenetic tree of cockatoos [17] indicates that Goffin’s cockatoos and White cockatoos are not particularly closely related, whereas Goffin’s cockatoo and little corella are reasonably closely related. Combined, our cluster analysis suggests that dance behaviour may not be connected to genetic similarities but instead similarities in dance behaviour between cockatoo species may arise due to other undetermined factors.

It is clear from our analysis of online videos that dance movements are like movements used in courtship displays. For example, the male red-tailed black cockatoo (*Calyptorhynchus banksia*) has been seen to land near a female and raise its crest with head bowed forward and proceed to rock from side to side and using short steps in a bowing motion move towards the female [18]. This series of movements is like a Downward, Side to Side and Sidestep dancing sequence. The similarity between courtship and captive movements in cockatoos may explain the source behaviour and development of non-courtship dancing behaviour. There is also the possibility that in the online videos birds were dancing to display courtship behaviour to their owners, a form of redirected behaviour. Keehn et al. [3] suggested that birds dancing may be a social behaviour used to interact with human caregivers. Although it appears reasonable that dancing behaviour may arise from courtship displays, our playback experiment somewhat refutes any role of the owners in eliciting dance behaviour. Further research is required to determine the motivational basis for this behaviour in captivity.

Studying dance behaviour in parrots may also provide useful models to understand complex cognitive processes in birds. Laland et al. [9] considers that dancing relies on a dancer’s ability to imitate because imitation plays an essential role in learning to dance and acquiring sequences of choreography that are dependent on social learning. Given the spontaneity and complexity of Snowball’s dance moves, Keehn et al. [3] argued that the proximity of the motor-learning regions with the vocal-learning regions in their forebrains might allow parrots to dance by improvising creative movements in sync with the music. Another interesting factor raised by our findings of 17 “rare” movements seen in only one bird is the possibility that dance may reflect creativity. Wiggins et al. [19] acknowledges that creativity is difficult to address from a scientific perspective in animals and proposes an approach for rational study of creativity is to consider the evolutionary perspective. For example, Kaufman et al. [20] reported that novel behavioural displays by male Australian satin bowerbirds (*Ptilonorhynchus violaceus*) significantly increase a male’s chance of acquiring a mate. Further research is required to determine whether rare dance movements may be unique to individual parrots and indicative of a creative process.

Dance behaviour may also provide some insights in the physiological and neurophysiological control of behaviour. Androgens are necessary for courtship displays and the motor neurons that innervate the wing muscles used for producing certain movements contain androgen receptors [21]. Fusani et al. [21] found androgens modulate courtship in the male golden-collared manakins (*Manacus vitellinus*) but the frequency of courtship dancing was not correlated with the concentration of circulation testosterone. Fuxjager and Schlinger [22] also found that testosterone increased at the onset of the breeding season and administering testosterone to non-breeding males increased courtship displays. Considering this connection of neuromuscular and androgenic hormonal systems involved in dancing, parrots may be activating these receptors through dancing. If dancing activates androgen receptors there would be a positive feedback effect that maintains the behaviour. Courtship behaviour may be the origin of the dance movements and it seems plausible that hormones that control courtship displays may also be involved in maintaining dancing behaviour in captivity.

One important consideration when observing dance behaviour is the possibility that the movements may be stereotypic. Stereotypic behaviour is defined as voluntary movement patterns without obvious function or goal, which are performed repeatedly and are relatively invariant in form [23]. Therefore, variation in movement patterns is important to differentiate dancing from potentially stereotypic behaviour. Parrots are particularly vulnerable to stereotypic behaviour which can include feather plucking, screaming and self-mutilation [24]. In general, we found considerable variation in dance movements to indicate that much of the dancing behaviour we observed was not stereotypic. Nonetheless, future research should include criteria for video inclusion to reduce the possibility of stereotypic behaviour being mis-classified as dancing behaviour. This could be supported by further research to understand dance movements, sequences and variation within and between individuals and species, and the development of a precise definition of dance behaviour that distinguishes these movements from stereotypies or agitation movements [25].

With respect to the implications for animal welfare, dance behaviour might fit the definition of play, as it is initiated voluntarily and appears intrinsically rewarding [26]. Parrots are kept in many captive settings such as zoos and as companion animals and their ethological needs are complex and relatively unchanged from their wild ancestors [24]. In contemporary animal welfare science, good welfare is not simply the absence of negative experiences but is also the presence of pre-validated, primarily positive experiences such as pleasure [27]. Within this context, play behaviour has attracted considerable attention due to the possibility it reveals positive welfare, and its encouragement through environmental enrichment or other management interventions may elicit positive responses. It is difficult to draw any conclusions on the welfare of the birds in the online videos, though the question of whether birds are experiencing pleasure in their dancing could provide an insight into understanding animal positive experiences.

Our findings from the playback experiment indicated that although dancing behaviour was observed in all six cockatoos in a zoo setting, music does not appear to be critical to trigger dancing. Dancing with no music was not due to a latent effect as the order of presentation of the treatments was changed across the nine days of treatment application. It is possible that 9 days across 6 weeks may not be enough exposure to the music to allow birds to develop a dance response to music. Alternatively, it is possible that motivation to interact with the opposite-sex partner may have overridden any attention and response to the playback. Additionally, the choice of song may not have been appropriate or repeated exposure to the same song may have impacted the birds’ response. Further research could determine the ability of music to trigger dancing and the ability of cockatoos to entrain to music using various beats and rhythms, though close attention to the properties of music and playback would support the validity and reliability of such research [28].

In conclusion, our findings provide further evidence that captive cockatoos commonly show dance behaviour that appears non-adaptive. Some aspects of dance behaviour in response to music appear to fit the definition of play and may thus indicate a positive welfare state. However, validation through independent positive welfare measures is still needed and care should be taken as similar movements may also indicate stereotypic behaviour. Dancing may also provide a useful paradigm to understand bird cognitive processes such as imitation, creativity and entrainment. We suggest that dance behaviour could be a valuable indicator of positive welfare and music playback could be a useful auditory enrichment. The preliminary playback study in Part 2 can be used as a stepping stone for further research and development of projects to better understand the dancing of parrots and how to trigger the behaviour for enriching purposes.

## Supporting information

S1 Table Table S1: Cockatoo videos used for data analysis in Part 1. Video numbers, URL, location of bird, duration of video and type of music are provided. (DOCX)

S2 Table Table S2: Previously defined and new cockatoo dance movements identified in our sample of 45 cockatoos with definitions, scoring method and examples. Footnote: *Existing movements from Keehn et al. [3], examples found in supplementary data 1-s2.0-S0960982219306049-mmc3.mp4 from https://www.sciencedirect.com/science/article/pii/S0960982219306049
(DOCX)

S3 Table Table S3: Rare cockatoo dance movements identified in only one bird. (DOCX)

S1 Fig Figure S1: Pie charts displaying the 10 most common movements in (a) Goffin cockatoos, (b) White cockatoos, (c) Sulphur crested cockatoos, (d) Moluccan cockatoos, and (e) Little corellas. (DOCX)

S2 Fig Figure S2: Number of different dance movements shown by 45 different parrots, from five species (x axis). (DOCX)

S3 fig Figure S3. Mean (se) probability of (a) single dance movements and (b) dance movements in a sequence occurring in response to Music playback, Podcast (white noise) and Control treatments of six cockatoos at Wagga Wagga zoo. (DOCX)
