# Supplementary material for: Dance behaviour in cockatoos: Implications for cognitive processes and welfare
Source: PLoS One. 2025 Aug 6;20(8):e0328487. doi: 10.1371/journal.pone.0328487 (PMC12327628; doi:10.1371/journal.pone.0328487)
Supplement: S1 Table — (DOCX) [file pone.0328487.s001.docx]

**Supporting Information**

Table S1: Cockatoo videos used for data analysis in Part 1. Video numbers, URL, location of bird, duration of video and type of music are provided.

| **video number** | **Species** | **URL** | **Music** | **Duration (min)** | **Location** | **Company** | **Comments** |
| --- | --- | --- | --- | --- | --- | --- | --- |
| 1 | White cockatoo (*Cacatua alba*) | <https://www.youtube.com/shorts/OF7KqL3LSaM> | Pop | 0.34 | Chair | 2 birds | One bird has bare patch on chest |
| 2 | White cockatoo | <https://www.youtube.com/watch?v=qTl1asCDOgs> | k-pop | 1.16 | Table | Alone |  |
| 3 | White cockatoo | <https://www.youtube.com/shorts/DoItzxy3eZw> | Pop | 0.35 | Couch | Alone |  |
| 4 | White cockatoo | <https://www.youtube.com/watch?v=CEQuDyuQFKE> | Rock | 2.26 | Chair | 2 birds | Human playing guitar and singing, one bird dancing other lifts foot |
| 5 | White cockatoo | <https://www.youtube.com/watch?v=sUVkdEEI-6o> | Kids pop | 1.07 | Chair | Humans | Human singing and cheering, bird vocalising |
| 6 | White cockatoo | <https://www.youtube.com/watch?v=YBnHIZKVQkM> | Rock | 1.42 | Cage | Alone | Human laughing |
| 7 | White cockatoo | <https://www.youtube.com/watch?v=1uDbABDAZ6s> | Rock | 1.0 | Chair | 2 humans | Human singing and laughing |
| 8 | White cockatoo | <https://www.youtube.com/watch?v=C3Jv_V_Yic0> | Accapella | 1.54 | Perch | Alone | Human singing tune to bird |
| 9 | White cockatoo | <https://www.youtube.com/watch?v=piwf-o9s_no> | Pop | 0.4 | Floor | Alone |  |
| 10 | White cockatoo | <https://www.youtube.com/watch?v=lh7TKAYwc0I> | Pop | 0.28 | Perch | Alone |  |
| 11 | White cockatoo | <https://www.instagram.com/p/BKUC2Y9AaYI/> | musical | 0.24 | Chair | Alone | Bird vocalising |
| 12 | White cockatoo | <https://www.instagram.com/p/BzqLZl_jQDM/> | Kids pop | 0.29 | Couch | Alone | Bird vocalising |
| 13 | White cockatoo | <https://www.youtube.com/watch?v=SAw4KXp_wUs> | kids tunes | 1.38 | Chair | Alone | Human singing alone with song |
| 14 | White cockatoo | <https://www.youtube.com/shorts/M6Z50k4cAIc> | rock | 0.41 | Table | 2 birds | One bird dancing from start, other bird joins in. |
| 15 | White cockatoo | <https://www.youtube.com/shorts/0Vd9bFr4fbg> | Pop | 0.18 | Perch | Alone | Bird vocalising |
| 16 | White cockatoo | <https://www.youtube.com/watch?v=zZq93T7Fbfw> | Rock | 1.06 | Perch and cage | Alone | Climbing around - low quality |
| 17 | White cockatoo | <https://www.youtube.com/shorts/Ek60G8sG2Ck> | Rock | 0.14 | Chair | Alone | Human laughing |
| 18 | White cockatoo | <https://www.youtube.com/watch?v=exIYiwbyJU4> | Pop | 0.27 | Perch | Alone | 3:56 - 4:23 |
| 19 | Sulphur crested cockatoo *(Cacatua galerita)* | <https://www.youtube.com/watch?v=c2lHtvKSxTc> | k-pop | 2.58 | Cage | Alone | Human laughing in background |
| 20 | Sulphur crested cockatoo | <https://www.youtube.com/watch?v=qw5CjzqK5Hw> | Pop | 1.06 | Floor | Alone |  |
| 21 | Sulphur crested cockatoo | <https://www.youtube.com/watch?v=5sSb1qF7m3w> | Pop | 1.09 | Couch | Alone | Bird vocalising |
| 22 | Sulphur crested cockatoo | <https://www.youtube.com/watch?v=5x3cvcQKJ7Y> | Rock | 0.19 | Legs | Human |  |
| 23 | Sulphur crested cockatoo | <https://www.youtube.com/watch?v=TqhFPdqBAmk> | Electronic | 1.27 | Table | Alone |  |
| 24 | Sulphur crested cockatoo | <https://www.youtube.com/watch?v=exIYiwbyJU4> | Raggae | 0.18 | Door | Alone | 1:57-2:15 |
| 25 | Sulphur crested cockatoo | <https://www.instagram.com/p/B4g8v3El4Yb/> | pop | 0.21 | Cage | Alone |  |
| 26 | Sulphur crested cockatoo | <https://www.youtube.com/watch?v=exIYiwbyJU4> | Pop | 0.28 | Perch | Alone | 3:28- 3:56 |
| 27 | Corella (*Cacatua sanguinea*) | <https://www.youtube.com/watch?v=0bt9xBuGWgw> | Rock | 2.42 | Perch | Alone |  |
| 28 | Corella | <https://www.youtube.com/watch?v=PDS56WtQJ4o> | Pop | 3.59 | Perch | Alone | Bird vocalising |
| 29 | Corella | <https://www.facebook.com/cara.jones1/videos/959650865676739> | Lyrical | 1.06 | Cage | 2 birds | Vocalising Corella is dancing, sulphur crested cockatoo is in neighbouring cage |
| 30 | Goffin cockatoo (*Cacatua goffiniana*) | <https://www.youtube.com/watch?v=qLyT4gQCFTc> | Pop | 1.23 | Table | 2 different species | vocalising, one bird dancing other watching, bare skin |
| 31 | Goffin cockatoo | <https://www.youtube.com/watch?v=exIYiwbyJU4> | Dance | 0.17 | Cage | 2 birds | 1:19-1:36, 1 in cage and one out of cage |
| 32 | Goffin cockatoo | [Polly the goffin cockatoo dancing to guns n ' roses (youtube.com)](https://www.youtube.com/watch?v=qBAnX8yhywM) | Rock | 2.22 | Perch | Alone | Bird vocalising |
| 33 | Goffin cockatoo | <https://www.youtube.com/watch?v=R-F4E8VQYXA> | Pop | 1.06 | Table | Alone | Bare skin on body and wings |
| 34 | Goffin cockatoo | <https://www.youtube.com/watch?v=5kgBUZlto8c> | Rap | 1.27 | Table | Alone | Video cuts from behind to front |
| 35 | Goffin cockatoo | <https://www.youtube.com/watch?v=4AOrlI4B8KY> | Rap | 2.49 | Floor | Alone | Human "whooping" |
| 36 | Goffin cockatoo | <https://www.youtube.com/watch?v=fyymIx-mqi4> | beatbox | 1.11 | Hand | Human | Bird vocalising, walking along hand |
| 37 | Goffin cockatoo | <https://www.youtube.com/shorts/rWbcpgj-uq8> | Pop | 0.11 | Shoulder | Human | Human talking |
| 38 | Goffin cockatoo | <https://www.tiktok.com/@pearldancetok/video/7108502046566780203?q=birds%20dancing&t=1709767549916> | Dance | 1 | Sink | Alone | Humans talking and encouraging |
| 39 | Goffin cockatoo | <https://www.tiktok.com/@lalalocokojo/video/6899584957228797186?q=birds%20dancing&t=1709767979763> | Metal | 0.56 | Cage | 2 birds |  |
| 40 | Goffin cockatoo | <https://www.youtube.com/shorts/th_QuGbQom0> | kids tunes | 0.15 | Shower | Alone | Human encouraging |
| 41 | Goffin cockatoo | <https://www.youtube.com/watch?v=qhi_qdshadE> | Bollywood | 0.26 | Chair | Alone | 1.34-2.0 |
| 42 | Goffin cockatoo | <https://www.youtube.com/watch?v=qhi_qdshadE> | Techno | 1.12 | Chair | Alone | 9.18-10.30 |
| 43 | Moluccan cockatoo (*Cacatua moluccensis*) | <https://www.youtube.com/watch?v=jzOJoLVkSTE> | Funk | 1.59 | Floor | Alone | Human walks past |
| 44 | Moluccan cockatoo | <https://www.youtube.com/watch?v=K1pE8ba55Gg> | Rap | 2.45 | Couch | Alone | Video of music playing in background, bird vocalising and climbs onto cage |
| 45 | Moluccan cockatoo | <https://www.youtube.com/watch?v=NSmiPBblVKE> | Rap | 0.52 | Table | 3 birds | 1 bird dancing (moluccan) and 2 watching (sulphur crested cockatoos) |
