# Supplementary material for: Dance behaviour in cockatoos: Implications for cognitive processes and welfare
Source: PLoS One. 2025 Aug 6;20(8):e0328487. doi: 10.1371/journal.pone.0328487 (PMC12327628; doi:10.1371/journal.pone.0328487)
Supplement: S2 Table — https://www.sciencedirect.com/science/article/pii/S0960982219306049 (DOCX) [file pone.0328487.s002.docx]

**Supporting Information**

Table S2: Previously defined and new cockatoo dance movements identified in our sample of 45 cockatoos with definitions, scoring method and examples.

| **Movement** | **Description** | **Scoring** | **Example** | **Notes** |
| --- | --- | --- | --- | --- |
| **Head** | | | | |
| Headbang (H)* | Head is flexed and extended rapidly | One headbang is head flexed and extended. | 0.35 |  |
| Downward (D)* | Head moving up and down (flexion/extension of head) | One count is one nod | 0.05 |  |
| Head Shake (HS) | Head flexion with rotation in synchrony | Head returns to upright position | 0.50 (6) |  |
| Side to Side (S)* | Head moves from side-to-side with rebound in neck | One count as head moves from left to right and returns to left. | 0.10 |  |
| Head Circle (HC)* | Head moves in a circular trajectory | Count when head returns to upright position | 0.56 (27) | Reported as counter-clockwise direction by Keehn et al (2019), but one cockatoo in our study moved head in both direction. |
| Head Downward and Shake (DS)* | Head shakes while head moving down | One count is one nod | 0.18 |  |
| Semi-Circle Low (SCL)* | Both feet remain close to or in contact w/ surface; head follows a semi-circle with minimal to none forward flexion | As head moves to the left | 0.47 |  |
| Semi-Circle High (SCH)* | Both feet remain close to or in contact w/ surface; head follows a semi-circle trajectory with minimal to none forward flexion | As head moves to the left | 0.50 |  |
| Head Turn (HT) | Head rotates side to side | One count is when head rotates and returns to forward facing (neutral) | 1.18 (28) |  |
| Head Figure 8 (HSW) | Head moves in a figure 8 configuration | One count as head returns to neutral position | 0.48 (8) |  |
| Crest | Added onto other movements with their definition + crest being raised | Counted the same as the original movement | 0.28 (9) | e.g. Downward *crest |
| **Wings** | | | | |
| Wings Back (WP) | Wings flexing and extending forward and back | Each time the wings are pushed in forwards position | 0.31(20) |  |
| Flapping (FW) | Rapid open and closing of wings | Counted as flapping bout when wings are open and flapping | 0.11(11) |  |
| Wings | Added onto other movements with their definition + wing abduction | Counted the same as the original movement | 0.28 (9) | e.g. Downward *wing |
| **Foot** | | | | |
| Foot-Lift (F)* | Foot lifts into air, body remains stationary | One lift and return to starting position | 0.23 |  |
| Sidestep (SS) | Foot steps to side and meets other foot | One count is foot to the side and meets other foot | 0.01(6) |  |
| **Foot and head combination** | | | | |
| Head-Foot Sync (HF)* | Head moves in sync with foot | Each time head is in forward position and foot is forward | 0.31 | Rare in current study |
| Foot-Lift Down Swing (FL)* | Foot lifts in combination with head turn and forward flexions while head swings diagonally downward | Head returns upright and foot returns to standing position | 0.27 | Rare in current study |
| Downward/Head-Foot Sync (D/HF)* | Head bobs up and down interspersed with head moving in sync with foot lift. | Head in downward position and foot lifted | 0.00 (34) |  |
| Downward Walk (DW) | Synchronised movement of head up and down while feet move side to side | As feet meet and head is in downward position | 0.24(6) |  |
| Sidestep with Side to Side (ST) | Sidestep combined with side flexion of head and body | One movement is as a sidestep and side flexion to the same side is complete | 0.46(31) |  |
| Headbang with Stepping (HBW) | Head moves forward and back while stepping forwards | Head in forwards position | 0.04(10) | Seen in one bird, scored as separate H and SS |
| **Whole body** | | | | |
| Body Roll (B)* | Wave passes through head, then body | One wave from start position and ends when head returns to start position | 0.58 |  |
| Pose (P)* | Body poses/holds a stationary position. Pose - variable | Count after bird changes to different movement | 0.44 |  |
| Fluff (FF) | Feathers are fluffed | Fluffing event | 0.49(6) |  |
| Stationary Jump (JS) | Jump up and down, both feet airborne and land in the same spot | Count as feet land back onto surface | 0.07(3) |  |
| Moving Jump (JM) | Jump sideways or forwards | Count as feet land back onto surface | 0.03(3) |  |
| Turn (T) | Steps around 180 degrees to face opposite direction | Once oriented in the opposite direction a turn is complete | 0.13(3) |  |
| Jump turn (JC) | Feet are airborne and bird jumps 360 degrees | As a full rotation is complete | 0.10(36) |  |
| Complex | Compound movement utilising 4 or more body parts which is not easily defined | Counted as one sequence and noted. | (5) 1.41  (7) 0.38  (37) 0.14 | A bout of complex movements |

Footnote: *existing movements from Keehn et al. (2019). “Example” column provides the time points where the movements are seen in the video from supplementary data 1-s2.0-S0960982219306049-mmc3.mp4 from <https://www.sciencedirect.com/science/article/pii/S0960982219306049> .
