# Supplementary material for: Dance behaviour in cockatoos: Implications for cognitive processes and welfare
Source: PLoS One. 2025 Aug 6;20(8):e0328487. doi: 10.1371/journal.pone.0328487 (PMC12327628; doi:10.1371/journal.pone.0328487)
Supplement: S3 Table — (DOCX) [file pone.0328487.s003.docx]

**Supporting Information**

Table S3: Rare cockatoo dance movements identified in only one bird.

| **Dance move** | **Video Number** | **Total count** |
| --- | --- | --- |
| Body roll with walk | 14 | 43 |
| Foot tap | 41 | 27 |
| Downward shake w/crest | 5 | 3 |
| Moving jump w/crest | 21 | 31 |
| Sidestep with side to side w/crest | 21 | 3 |
| Downward w/wing | 24 | 4 |
| Sidestep with side to side w/wings | 24 | 27 |
| Semi-circle low w/crest | 27 | 2 |
| Head figure 8 w/wings | 30 | 4 |
| Head counter-clockwise Circle w/wings | 33 | 2 |
| Downward shake w/wings | 35 | 6 |
| Jump turn w/wings | 36 | 35 |
| Moving jump w/wings | 36 | 29 |
| Stationary jump w/wings | 36 | 5 |
| Semi-circle low w/wings | 37 | 17 |
| Head figure 8 w/crest | 40 | 9 |
| Semi-circle high w/wings | 43 | 4 |
