# Supplementary material for: Dance behaviour in cockatoos: Implications for cognitive processes and welfare
Source: PLoS One. 2025 Aug 6;20(8):e0328487. doi: 10.1371/journal.pone.0328487 (PMC12327628; doi:10.1371/journal.pone.0328487)
Supplement: S1 Fig — (DOCX) [file pone.0328487.s004.docx]

**Supporting Information**

Figure S1: Pie charts displaying the 10 most common movements in (a) Goffin cockatoos (N=13), (b) White cockatoos (N=18), (c) Sulphur crested cockatoos (N=8), (d) Moluccan cockatoos (N=3), and (e) Little corellas (N=3).

(a)

**
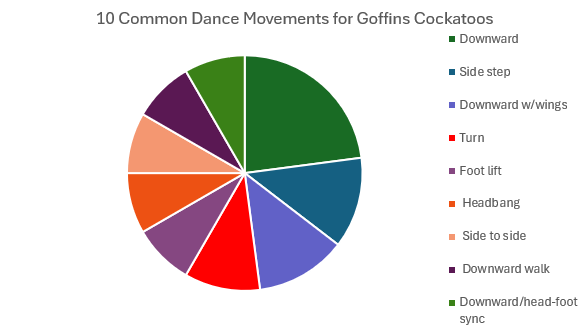
**

(b)

**
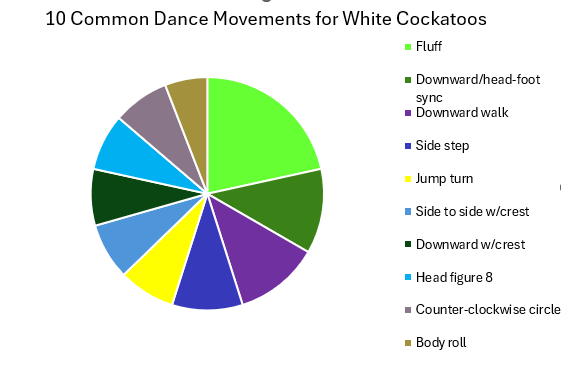
**

(c)

**
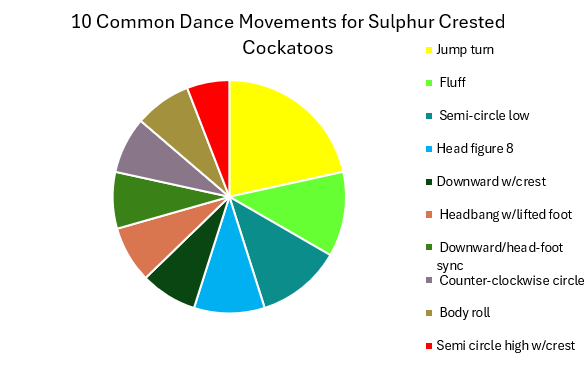
**

(d)

**
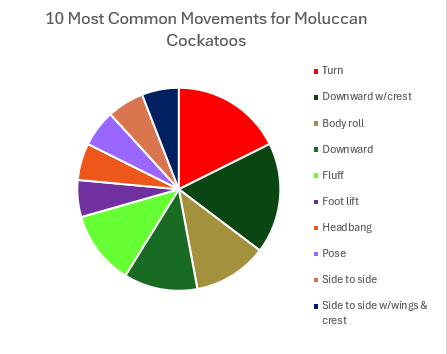
**

(e)

**
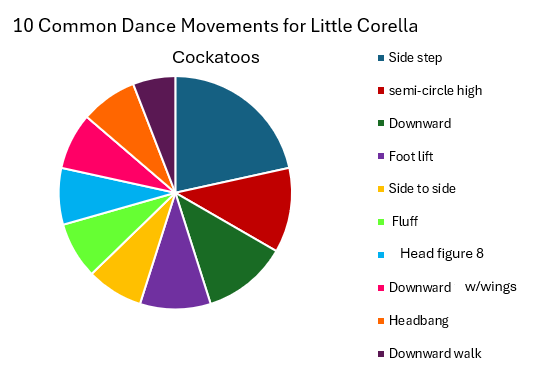
**
