# Supplementary material for: Dance behaviour in cockatoos: Implications for cognitive processes and welfare
Source: PLoS One. 2025 Aug 6;20(8):e0328487. doi: 10.1371/journal.pone.0328487 (PMC12327628; doi:10.1371/journal.pone.0328487)
Supplement: S2 Fig — (DOCX) [file pone.0328487.s005.docx]

Figure S2: Number of different dance movements shown by 45 different parrots, from five species (x axis).
