# Supplementary material for: Dance behaviour in cockatoos: Implications for cognitive processes and welfare
Source: PLoS One. 2025 Aug 6;20(8):e0328487. doi: 10.1371/journal.pone.0328487 (PMC12327628; doi:10.1371/journal.pone.0328487)
Supplement: S3 fig — (DOCX) [file pone.0328487.s006.docx]

**Supporting Information**

Figure S3. Mean (se) probability of (a) single dance movements and (b) dance movements in a sequence occurring in response to Music playback, Podcast (white noise) and Control treatments of six cockatoos at Wagga Wagga zoo.

(b)
